# Ether as an Anæsthetic Agent

**Published:** 1860

**Authors:** 


*Ether as an Anæsthetic Agent.*

The more anæsthesia is employed in surgery, and the wider its application becomes in practical medicine and obstetrics, the more do we realize the terrible results which may arise from the administration of chloroform, and the immense superiority of ether as anæsthetic agent, so far as regards the safety of the patient. We are gratified to see that this subject is attracting more and more the attention of the profession. We now hardly think a surgeon has a right to administer chloroform, when so many cases of death are being reported every day, and these in the most skillful and experienced hands. Of all agents for producing insensibility, we believe that ether is the most free from danger, and, if pure, it can almost be said that it is absolutely safe.

As a general rule, the lower animals are more susceptible to the influence of anæsthetics than the human subject; and all who have had occasion to use these agents upon them to any extent must be convinced that chloroform is exceedingly unsafe. So much so, indeed, that we are about as likely to kill the animal with chloroform, even when given with the greatest care, as to administer it successfully. We have had occasion to operate this winter, however, upon dogs and cats, always putting them under the influence of ether, and have been surprised several times by the unexpected death of the animal. This has been entirely unexpected, for we have been able to administer ether with the greatest freedom, and, indeed, have found it difficult to kill an animal with it, even when we desired to do so. This circumstance we have explained by the impurity of the ether; and we think that we can learn an important lesson from this in our administration of ether to the human subject. But one or two cases of death from the administration of ether in the human subject, are upon record. One of them was presented by Dr. Alonzo Clark, to the Pathological Society some months ago, and was published with the Proceedings in this Journal. In this case, our readers will remember, post-mortem examination disclosed disease of the cerebellum, which made it not a fair case of death from the effects of the ether. Though deaths from ether so seldom occur, we see by the effects of impure ether upon the lower animals, that a bad article is much more dangerous than a good one, and that it is of the greatest importance that this article should be perfectly pure. It has long been held that death from chloroform only takes place as a result of the impurity of the agent; and its advocates have contended that when pure it was perfectly safe. That the former is not the case, has now been abundantly proven; though an impure article is much more dangerous than the pure. Sufficient attention has not, we think, been paid to the quality of the ether used for anæsthetic purposes; a point which we are convinced should not be overlooked.—*N. Y. Monthly Review.*

---

We are pleased to perceive that the journals are beginning to speak out more freely in reference to the use of chloroform. If it be true that it is *doubtful*, as suggested above, whether a *Surgeon* is justifiable in administering chloroform when so many cases of death are being reported every day, and *these in the most skillful and experienced hands*, what shall be thought of those who unhesitatingly administer this agent upon such occasions as the *extraction of teeth*, or to relieve the suffering connected with *natural labor*, and who to meet the whims and caprices of their patients, or *to secure patronage*, are willing to forget their solemn responsibilities and flippantly and foolishly declare, in the face of the most incontrovertible facts, that there is no danger.

With the present lights before us, and a knowledge of the fact that so large a number ot individuals have died from the effects of the most skillful administration of chloroform, and in some instances from very small quantities, and that too in cases where the individual had previously taken it without serious results, it becomes a question whether the physician who wilfully or ignorantly disregards these fearful warnings, and should be so unfortunate as to meet with a fatal case, is not guilty of the *crime of murder*, and should not be punished as such by the laws of the land.

